# Improving Equity of Care for Culturally and Linguistically Diverse Patients: Utilising Multilingual Software to Support Breath‐Hold Breast Radiation Therapy

**DOI:** 10.1002/jmrs.70095

**Published:** 2026-06-10

**Authors:** Toby Lowe, Rory Hartley, John Atyeo, Brooke Griffiths, Brian Porter, Gillian Lamoury, Marita Morgia, Gabrielle Metz, Susan Carroll

**Affiliations:** ^1^ Department of Radiation Oncology, Northern Sydney Cancer Centre Royal North Shore Hospital Sydney New South Wales Australia; ^2^ Sydney Medical School, University of Sydney Sydney New South Wales Australia

## Abstract

Breath‐hold techniques, including Deep Inspiration Breath Hold (DIBH) and Exhalation Breath Hold (EBH), are commonly used in radiation therapy to reduce radiation exposure to organs at risk while maintaining accurate tumour targeting. However, high failure rates during breath‐hold simulations have been associated with patient anxiety and insufficient preparation—particularly when instruction is given only 20 min prior to the procedure. These challenges are amplified for patients from culturally and linguistically diverse (CALD) backgrounds. To address this, a multilingual mobile application (app) has been developed to improve patient education, communication, and compliance, with a particular focus on supporting CALD patients. Replacing traditional pamphlets with interactive instructional videos, visual aids, and real‐time translation in seven languages, the app allows patients to familiarise themselves with breath‐hold techniques before their Computed Tomography (CT) simulation. A General Radiation Therapy (RT) section in the app further supports communication between radiation therapists (RTs) and their patients by providing translated commands for standard patient positioning and movement instructions, improving accuracy and workflow efficiency. The app integrates with in‐room sound systems, allowing RTs to provide remote coaching while maintaining clear and consistent communication. Additionally, the Frequently Asked Questions (FAQ) section of the app addresses common concerns that patients have about radiation therapy, helping them gain a better understanding with the possibility of reducing anxiety. The app has been implemented at the Northern Sydney Cancer Centre (NSCC) and is expected to improve patient compliance in breath‐hold techniques and streamline clinical workflows.

## Graphical abstract

A 22% breath‐hold failure and exclusion rate highlighted major communication barriers for CALD breast radiotherapy patients. In response, a multilingual app was developed to deliver translated coaching for DIBH and EBH in seven languages, improving health equity within the local health district through early education, patient‐friendly FAQs, and clearer communication.

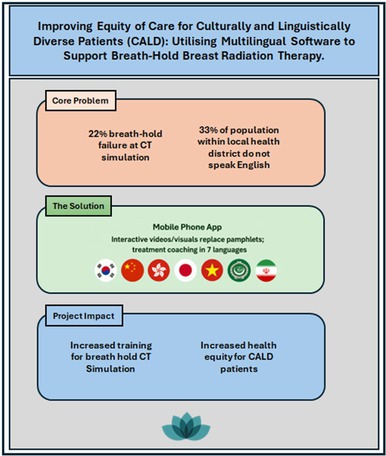

## 1 Introduction

Advancements in breast cancer screening have enabled earlier detection, leading to significantly improved treatment outcomes [1]. Radiation therapy is integral to the multidisciplinary management of early breast cancer, both for patients undergoing breast‐conserving surgery and for select patients following mastectomy. Whole breast irradiation is conventionally administered using tangential intensity‐modulated radiation therapy (IMRT) or volumetric modulated arc therapy (VMAT), both of which are designed to achieve a homogenous dose distribution within the target volume. Nonetheless, despite these technological advancements, interfractional target motion can result in dose inhomogeneity. This variability may adversely affect cosmetic outcomes and, in certain cases, compromise local tumour control [2, 3, 4, 5, 6, 7].

To mitigate motion‐related uncertainties and reduce radiation exposure to organs at risk (OARs), breath‐hold techniques during radiation therapy have become established practices. These approaches allow for more precise targeting by synchronising radiation delivery with specific phases of the respiratory cycle. Deep inspiration breath hold (DIBH) is the most widely adopted of these techniques, particularly for patients with left‐sided breast cancer, due to its proven ability to significantly reduce cardiac dose [8, 9, 10, 11]. A range of DIBH methods are available, from simple voluntary breath holds to more advanced systems using spirometry or external surrogates [8, 9, 10, 11]. In addition to cardiac sparing, breath‐hold techniques have also demonstrated reductions in lung dose for both left‐ and right‐sided treatments [10, 12].

While DIBH is generally well tolerated, variability in diaphragmatic position during inspiration may limit its reproducibility in some patients. For treatment sites situated closer to the diaphragm, such as the lower lungs, liver, pancreas, stomach, or distal oesophagus, expiration breath hold (EBH) may offer improved stability of the target volume [13]. By minimising respiratory motion, breath‐hold techniques allow for tighter planning margins and potentially reduce incidental radiation to surrounding healthy tissues [13, 14, 15].

Within the Northern Sydney cancer Cente (NSCC), approximately 33% of patients are identified as culturally and linguistically diverse (CALD), with an estimated 12% reporting no English proficiency. The predominant languages represented in this cohort include Mandarin, Cantonese, Korean, and Japanese. At the NSCC, 11% of CALD patients were unable to undertake breath‐hold techniques owing to an inability to comprehend the verbal instructions delivered by radiation therapists. As a result, these patients received free‐breathing radiation therapy for their breast treatment, despite the established dosimetric and clinical benefits associated with breath‐hold techniques. Although the integration of interpreters into Computed Tomography (CT) simulation and treatment workflows has been shown to be feasible, evidence indicates that such models require substantial resourcing, extended appointment times, and incur additional financial and operational burdens [16].

The success of breath‐hold techniques relies heavily on patient training and engagement. Patients must be able to consciously regulate their breathing across multiple treatment fractions to ensure accurate dose delivery. Latty et al. [17], demonstrated that patient compliance is typically high; however, preparation and training protocols vary significantly between institutions. While many centres incorporate breath‐hold instruction prior to CT simulation, few clearly distinguish these preparatory sessions from the simulation itself. This highlights the need for standardised training practices, improved staff guidance, clearer patient selection criteria, and robust verification workflows to enhance the effectiveness and efficiency of breath‐hold techniques [17, 18].

At the NSCC, all patients are evaluated for their suitability to undergo breath‐hold techniques based on their respiratory function and ability to follow procedural instructions. Patients who are unable to meet the necessary criteria are treated under free‐breathing conditions.

This report presents the development and implementation of a mobile application (app) aimed at improving access to breath‐hold radiation therapy techniques for CALD patients. By enhancing patient education, training, and compliance, the app seeks to support more effective and accessible treatment delivery for this patient group.

## 2 Software Development and Format

To enhance patient education and improve access to breath‐hold techniques for CALD patients, a dedicated multilingual software app—available to both patients and staff on Android and Apple devices—has been developed and introduced into clinical practice. Created and coded by Lokava Software (Sydney, NSW), the app is designed to provide comprehensive education about DIBH and EBH techniques, aiming to reduce patient anxiety and improve breath‐hold performance. The app offers instructional videos, visual aids and instructions in multiple languages, ensuring that patients from CALD backgrounds have equal access to the same high‐quality information. All translations are closed loop and fixed within the platform to preserve linguistic integrity and consistency; they cannot be modified during delivery. Each translation has undergone review and validation in collaboration with New South Wales Health consumer groups to ensure linguistic accuracy, cultural appropriateness, and alignment with health literacy standards.

The development and evaluation process additionally involved consultation with the health district's consumer advisory group and targeted engagement with CALD patient representatives to assess usability, acceptability, and appropriateness. By providing these resources prior to CT simulation, the app familiarises patients with the breath‐hold procedure, thereby enhancing procedural understanding, confidence, and compliance. Through this approach, all patients—irrespective of language proficiency—receive standardised, evidence‐based instruction to support safe and effective delivery of breath‐hold radiation therapy.

## 3 Patient Selection

For patients to be eligible for the workflow with the software, they must be 18 years or older with a confirmed breast cancer diagnosis who are receiving radiation therapy at the NSCC. The workflow applies to those requiring a breath‐hold technique as part of their clinically indicated treatment plan and who have completed a planning CT scan. Patients without access to a smartphone or whose primary language is not among the seven supported by the mobile app—English, Cantonese, Mandarin, Japanese, Arabic, Vietnamese, and Korean—are not included in this workflow.

## 4 Patient Education

A data‐mining review of an 18‐month period showed that 11% of patients were unable to comply with the required DIBH or EBH breath‐hold technique during their CT simulation for breast cancer. An additional 11% of patients from CALD backgrounds were excluded from using breath‐hold techniques due to difficulty understanding the verbal instructions provided by radiation therapists. Consequently, all these patients received free‐breathing radiation therapy for their breast treatment despite the known clinical benefits of breath‐hold approaches. Ethics approval for ongoing data mining from the NSCC Research Databank was previously granted by the Human Research Ethics Committee of the NSCC (HREC reference 2019/ETH08469).

Post‐treatment patient surveys identified the timing of education as a contributing factor. Education was traditionally delivered only 20 min before the simulation appointment, and many patients reported that receiving information so close to the procedure increased anxiety and stress, reducing their ability to perform breath‐hold techniques successfully. It is hypothesised that this delayed education contributed to the observed 11% failure rate during CT simulation.

**FIGURE 1:**
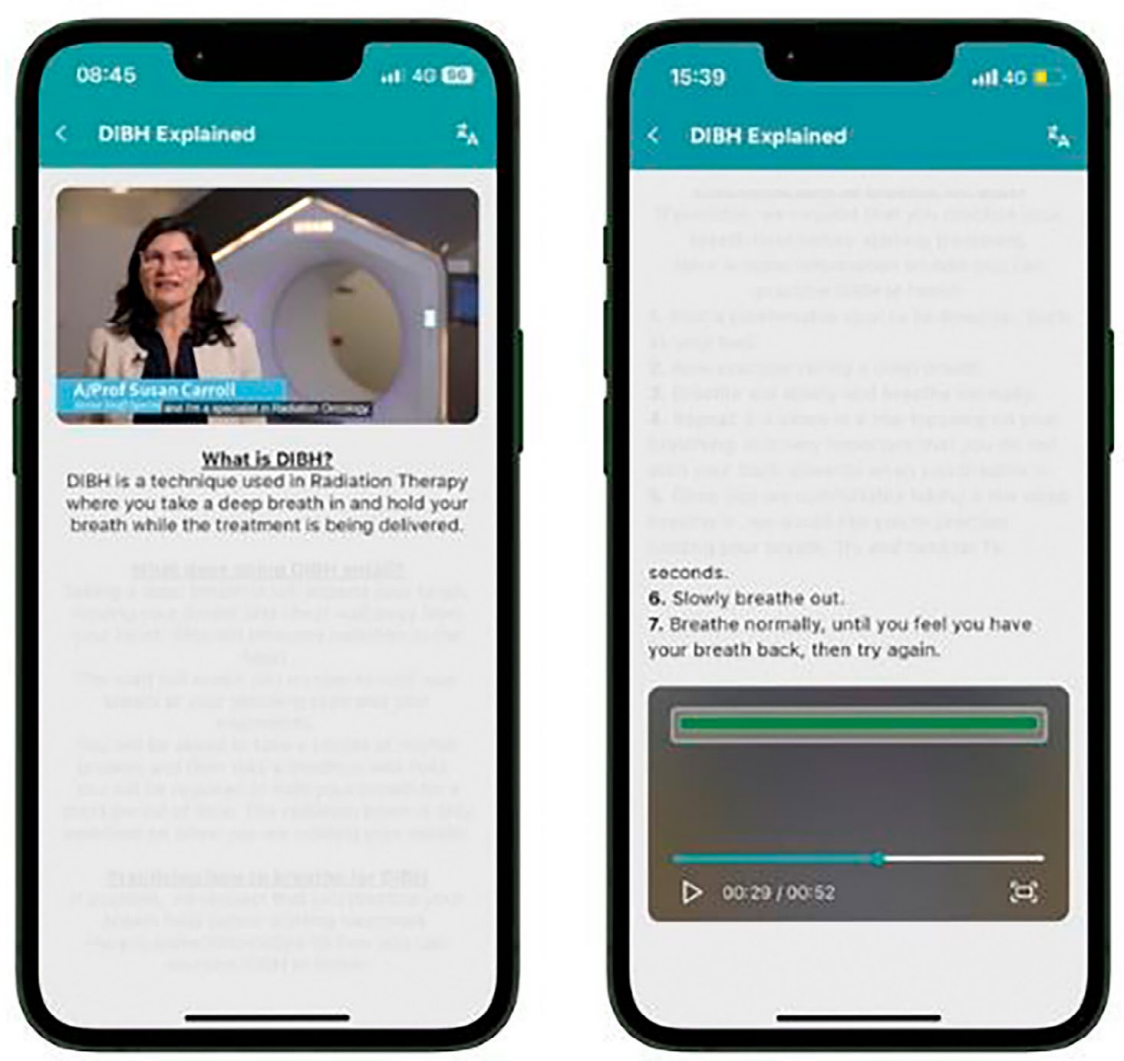
Screenshot of an educational page in English for DIBH.

To address this issue of failure rate, the existing patient education—previously delivered through an English‐only pamphlet—was incorporated into dedicated multilingual software. This digital platform provides the same detailed information and additionally includes an educational video outlining the CT simulation process (see Figure 1). The video offers visual guidance on key components of simulation and treatment, including patient positioning, the anatomical benefits of breath‐hold techniques such as DIBH and EBH, and an overview of what patients can expect during both CT simulation and treatment. The content is designed to be accessed independently by patients in their own time in one of seven languages.

Additionally, to further enhance patient preparation, a visual‐aid video was developed to replicate the experience of viewing the monitor used during CT simulation. This video incorporates verbal instructions that guide the patient through each step of the process. By familiarising patients with both the visual cues they will observe and the auditory commands they will hear, the resource aims to reduce uncertainty and improve adherence to breath‐hold instructions.

**FIGURE 2:**
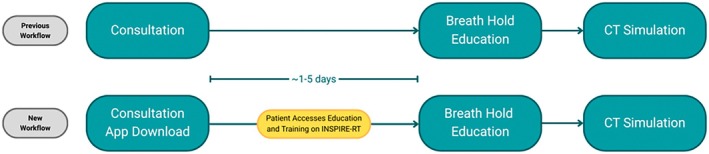
New workflow for training/education prior to CT simulation.

All educational materials—including the digital pamphlet, instructional video, and visual‐aid video—are available in seven languages, ensuring accessibility for a diverse patient population. By providing this comprehensive multilingual education package up to five days prior to CT simulation (see Figure 2), the aim is to reduce patient anxiety, support compliance, and ultimately improve the success rate of breath‐hold simulations for radiation therapy.

To maintain consistency of care, all patients continue to attend a breath‐hold education appointment, during which they can re‐watch the educational videos and ask questions before their CT simulation scan. A radiation therapist is present during this session to provide clarification and support.

## 5 Treatment Translation

**FIGURE 3:**
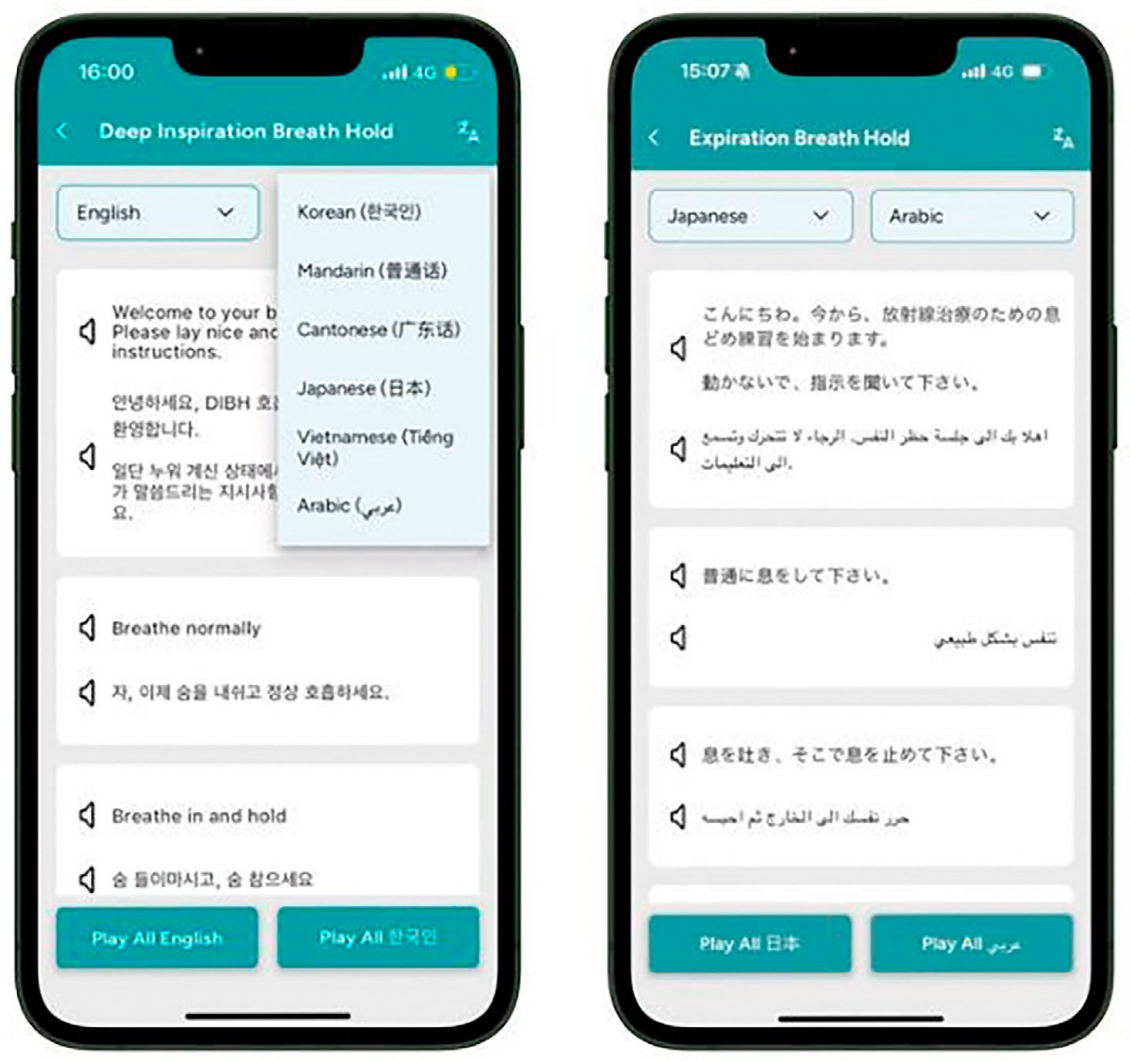
English to Korean and Japanese to Arabic translations for DIBH.

The treatment section provides consistent and clear translated DIBH and EBH commands, allowing seamless communication based on the patient's chosen language. This can be seen in Figure 3 which presents an example of English translated into Korean and Japanese translated into Arabic. This section consists of ten structured phrases designed to support RTs throughout the treatment process. These phrases include greeting the patient, confirming critical clinical information such as the correct patient procedure site (CPPS), and guiding the patient through the breath‐hold window while using the visual aid for additional support.

To enhance communication and ensure patients receive clear and consistent instructions, the device running the app is integrated with the in‐room sound system. This setup allows RTs to coach patients remotely from outside the treatment room and maintain verbal guidance throughout the procedure. Alternatively, the device can be used in direct conversation with the patient inside the treatment room during the setup process. Carrying the device into the room enables RTs to provide real‐time translated instructions and reinforcement, helping patients understand positioning requirements and breath‐hold expectations.

Interpreters continue to be utilised at key stages of the patient pathway, including during consultations with the radiation oncologist for personalised discussions, and at CT simulation when requested by the clinical team. Within the treatment environment, RTs are able to communicate accurately and consistently, as all translations are provided according to the patient's language preference and delivered in the selected language, as shown in Figure 3.

Prior to implementation, staff were trained in the use of the software through dedicated in‐services and supported by formalised protocols to ensure consistent practice. By incorporating in‐room sound system integration, flexibility for direct interaction, structured staff training, and interpreter support where appropriate, the app enhances the efficiency of the treatment process while reducing potential language barriers.

## 6 General Radiation Therapy Section

The software is designed for use directly in the treatment room, allowing RTs to bring a tablet or mobile phone to the treatment couch to assist with patient setup. This enables real‐time delivery of translated commands while visually guiding patients into the correct position for DIBH, EBH, or standard setup of patients not undergoing breath‐hold techniques.

The General Radiation Therapy section provides structured, clinically relevant phrases that support RTs in delivering consistent messaging through key stages of the setup process. These include greeting the patient, confirming important clinical details such as CPPS, and giving clear instructions for positioning. Commands such as “Please lie still,” “Move up the bed slightly,” and “Place your arms like this” help RTs efficiently correct and refine the patient's setup on the couch.

The section also contains translated instructions that prepare patients for treatment, such as “We are about to begin,” “You may hear a sound from the machine,” and “Let us know if you feel uncomfortable”, helping patients remain informed, reassured, and cooperative throughout the procedure.

In addition to use on the couch, the app can be connected to the in‐room audio system if remote coaching is required. By providing consistent, accessible commands in multiple languages, the General RT section enhances patient understanding, supports accurate patient setup, and contributes to a smoother, more efficient workflow for RTs.

## 7 Frequently Asked Questions (FAQ) Section

A Frequently Asked Questions (FAQ) section in the app was designed to provide patients with clear and reliable information about radiation therapy. This section draws from commonly asked questions sourced from the Cancer Institute NSW [19] and resources provided by the NSCC, ensuring that patients receive accurate and up‐to‐date responses. Each question and response has been translated into all seven supported languages, making the information accessible to a diverse patient population.

**FIGURE 4:**
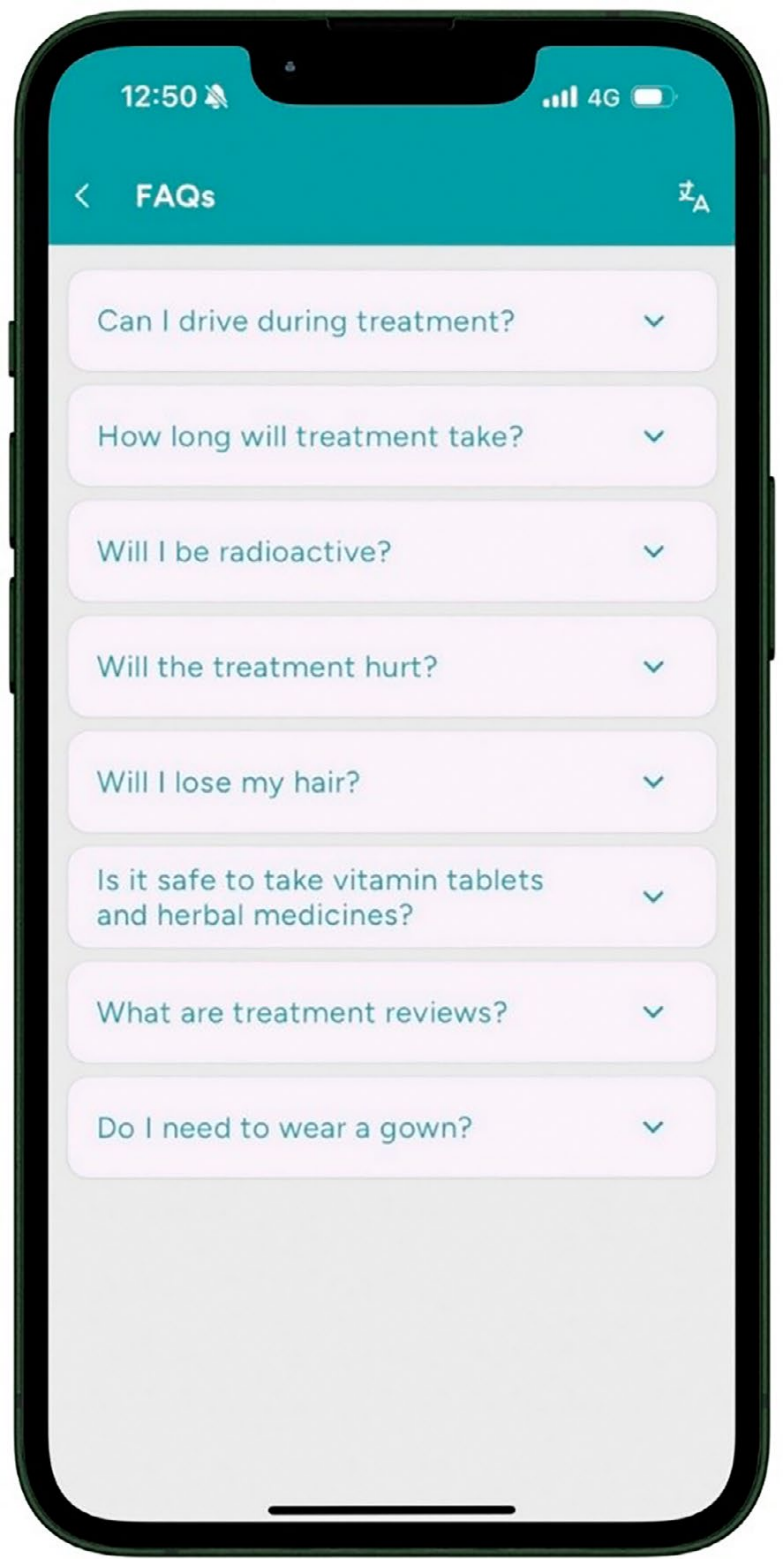
Frequently Asked Questions.

The FAQ section addresses various concerns that patients may have before and during their treatment. It explains the typical duration of daily radiation therapy sessions and the factors that may affect appointment length. Patients are also informed about treatment reviews, which involve regular check‐ins with their care team to assess progress and manage any side effects or concerns. Information is also provided on potential side effects, dietary recommendations, and post‐treatment expectations (Figure 4 example in English).

In addition to these core topics, the FAQ section includes practical information such as details about patient parking for radiation therapy appointments, ensuring patients can plan their visits with ease. It also provides an avenue for ongoing improvement by allowing space for additional feedback from both staff and patients. This feedback helps enhance the app's functionality and ensure the content remains relevant and helpful. All Supporting Information, like the primary FAQ content, is fully translated into the seven supported languages to maintain accessibility for all patients.

## 8 Future Directions

The current integration of the software supports Breast DIBH education and treatment delivery, with content available in seven languages, including English. Future development will focus on expanding the platform to additional tumour streams, including genitourinary and gynaecological pathways, where patients require clear guidance on bladder and rectal preparation. Expansion into central nervous system (CNS) pathways is also planned, with dedicated modules to educate patients on the importance of mask fabrication and immobilisation to support treatment accuracy.

There is also scope to enhance multidisciplinary engagement, particularly for head and neck cancer, through collaboration with allied health disciplines such as speech pathology, dietetics, nursing and physiotherapy. This approach aims to provide patients with a single, comprehensive resource that integrates education and support across the entire radiation therapy journey.

To evaluate the clinical impact of this integration, a series of patient and staff surveys is planned. These will assess patient experience, staff usability, and health economic outcomes. With the expansion into genitourinary streams, there is also a future opportunity to measure the impact of the software on patient compliance with bladder‐filling and rectal‐preparation protocols.

## 9 Conclusion

The multilingual app represents a significant advancement in supporting CALD patients undergoing breath‐hold radiation therapy techniques such as DIBH and EBH, and patient set‐up in general. By addressing key challenges at the NSCC, including the limitations of pamphlet‐based education and the absence of accessible, early‐stage information, the app strengthens CALD patients' ability to understand and engage in their treatment.

Through instructional videos, visual aids, and real‐time translation across seven languages, the platform delivers clear and culturally appropriate education tailored to diverse communication needs.

Ongoing evaluation will inform future enhancements, including additional languages and more targeted information for expansion into other tumour streams. This initiative reflects a proactive commitment to building more inclusive and equitable radiation therapy pathways for CALD communities.

## Funding

The authors have nothing to report.

## Conflicts of Interest

The authors Toby Lowe and Rory Hartley are co‐owners of the intellectual property rights for the INSPIRE‐RT application described in this manuscript. The remaining authors have no conflicts of interest to declare.

## Data Availability

The data that supports the findings of this study are available in the Supporting Information of this article.
